# Chemotaxis in Escherichia coli: A Molecular Model for Robust Precise Adaptation

**DOI:** 10.1371/journal.pcbi.0040001

**Published:** 2008-01-04

**Authors:** Clinton H Hansen, Robert G Endres, Ned S Wingreen

**Affiliations:** 1 Department of Physics, Princeton University, Princeton, New Jersey, United States of America; 2 Department of Molecular Biology, Princeton University, Princeton, New Jersey, United States of America; University of California San Diego, United States of America

## Abstract

The chemotaxis system in the bacterium Escherichia coli is remarkably sensitive to small relative changes in the concentrations of multiple chemical signals over a broad range of ambient concentrations. Interactions among receptors are crucial to this sensitivity as is precise adaptation, the return of chemoreceptor activity to prestimulus levels in a constant chemoeffector environment. Precise adaptation relies on methylation and demethylation of chemoreceptors by the enzymes CheR and CheB, respectively. Experiments indicate that when transiently bound to one receptor, these enzymes act on small assistance neighborhoods (AN) of five to seven receptor homodimers. In this paper, we model a strongly coupled complex of receptors including dynamic CheR and CheB acting on ANs. The model yields sensitive response and precise adaptation over several orders of magnitude of attractant concentrations and accounts for different responses to aspartate and serine. Within the model, we explore how the precision of adaptation is limited by small AN size as well as by CheR and CheB kinetics (including dwell times, saturation, and kinetic differences among modification sites) and how these kinetics contribute to noise in complex activity. The robustness of our dynamic model for precise adaptation is demonstrated by randomly varying biochemical parameters.

## Author Summary

Bacteria swim in relatively straight lines and change directions through tumbling. In the process of chemotaxis, a network of receptors and other proteins controls the tumbling frequency to direct an otherwise random walk toward nutrients and away from repellents. Receptor clustering and adaptation to persistent stimuli through covalent modification allow chemotaxis to be sensitive over a large range of ambient concentrations. The individual components of the chemotaxis network are well characterized, and signaling measurements by fluorescence microscopy quantify the network's response, making the system well suited for modeling and analysis. In this paper, we expand upon a previous model based on experiments indicating that the covalent modifications required for adaptation occur through the action of enzymes on groups of neighboring receptors, referred to as assistance neighborhoods. Simulations show that our proposed molecular model of a strongly coupled complex of receptors produces accurate responses to different stimuli and is robust to parameter variation. Within this model, the correct adaptation response is limited by small assistance-neighborhood size as well as enzyme kinetics. We also explore how these kinetics contribute to noise in the chemotactic response.

## Introduction

Through the process of chemotaxis, the bacterium Escherichia coli swims up the concentration gradients of attractants (nutrients) and down the concentration gradients of repellents. E. coli moves via the rotation of multiple flagella. When the flagella rotate counterclockwise, they bundle and propel the bacterium forward. Rotation in a clockwise direction causes the flagella to fly apart, and the organism tumbles to change direction. Swimming up a gradient of attractant causes a decrease in the probability of tumbling, whereas swimming up a gradient of chemorepellent causes an increase in the probability of tumbling. The result is that E. coli performs a biased random walk toward chemoattractants and away from chemorepellents [1].

The signaling pathway that governs E. coli chemotaxis is well characterized [2–4]. Out of five different membrane-bound chemotaxis receptors, Tar and Tsr are expressed at high levels, whereas Tap, Trg, and Aer are expressed at lower levels. The receptors form homodimers that can each bind one molecule of ligand [5]. The homodimers in turn form trimers of dimers, and associate with CheW and CheA. CheW is a linker protein, and CheA is a histidine kinase [6,7]. Receptor signaling activates CheA autophosphorylation, and the phosphoryl group is transferred to the response regulator, CheY. Phosphorylated CheY diffuses and binds to the flagellar motors, favoring clockwise rotation and tumbling. CheY is dephosphorylated by the phosphatase CheZ.

E. coli are able to react to small relative changes in concentration over a range of several orders of magnitude. In experiments done by Mao et al. [8], bacteria responded to changes in concentration from 10 mM to as low as 3.2 nM of the attractant aspartate. Two properties of the network that underlie the broad range of responsiveness are interactions among receptors and precise adaptation [9]. In vivo fluorescence resonance energy transfer (FRET) measurements [10,11] suggest that signaling is mediated by strongly coupled complexes of 10–20 receptor homodimers that are all active or inactive together. FRET also reveals that levels of phosphorylated CheY adapt precisely following a transient response to steps of chemoeffector concentration. Precise adaptation occurs though the methylation by CheR and demethylation by CheB of eight sites on each homodimer receptor [12,13]. Methylation at each site increases the activity level of receptors, whereas demethylation decreases activity. Each Tar or Tsr receptor has a tether at its C terminus, with a pentapeptide site that can bind one CheR or CheB [14]. Experiments indicate that when transiently bound to one receptor, each CheR or CheB can act on five to seven adjacent receptor homodimers, defining an “assistance neighborhood” (AN) [15].

The dynamics of receptor modification in complexes is not well understood. A two-state single-receptor model was proposed by Barkai and Leibler in which the modification activities of CheR and CheB depend only on receptor activity, not ligand concentration or methylation level [16]. This simple model naturally leads to precise adaptation, but does not include interactions among receptors. More recent approaches have incorporated receptor interactions using a Monod–Wyman–Changeux (MWC) model [17] in which a complex of receptors is either active (on) or inactive (off) as a whole [11,18–20]. The free-energy difference between the on and off states of a complex dictates the probability of its being in the active state. To preserve precise adaptation within the MWC model, the Barka–Leibler (BL) model was extended [18,19] to include the action of CheR/CheB on ANs of receptors. Static nonoverlapping ANs of size 6 were utilized. Here, we build on this earlier model by incorporating the binding and unbinding of CheR and CheB, creating dynamic ANs. This extension allows us to consider limits to precise adaptation from small AN size as well as from CheR and CheB kinetics, including dwell times, saturation, and kinetic differences among modification sites.

Models for the E. coli chemotaxis network are complex and depend on numerous parameters, bringing into question how well the essential property of precise adaptation is preserved when parameters are altered. Barkai and Leibler showed that their simple two-state single-receptor model of the chemotaxis network was robust to parameter variation [16]. We find a similar robustness of our dynamic AN model.

## Model

### Complex Activity

In this paper, we explore a MWC model of mixed and strongly coupled Tar and Tsr chemoreceptor homodimers. Within the MWC model, a complex of receptors is either on or off as a whole (Figure 1). The average complex activity is the probability that the complex is in the on state and is determined by the free-energy difference between the on and off states [19]. Here, the MWC model is used to calculate the thermal equilibrium complex activity from the instantaneous attractant concentration and receptor methylation state.

**Figure 1:**
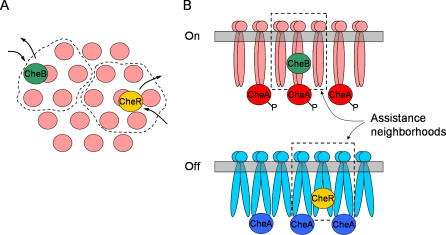
Two-State Receptor Complex and Precise-Adaptation Model (A) Top view of the hexagonal arrangement of the 19-receptor homodimer used in our simulations. Each receptor can bind to either CheR or CheB. Each bound CheR or CheB can then act on an “assistance neighborhood” of adjacent receptors (dashed line) through methylation (CheR) or demethylation (CheB). (B) Side view of receptor complex in the on state (top) and off state (bottom) with, respectively, active and inactive receptor-bound kinases CheA. Active CheA kinases autophosphorylate to CheA-P. In our adaptation model, CheB only demethylates receptors when the complex is in the on state (top), and CheR only methylates receptors when the complex is in the off state (bottom).

We assume that each receptor homodimer is a two-state system, being either on or off. Each receptor homodimer can bind a ligand molecule in either state, albeit with different affinities. Therefore, the four possible configurations for each homodimer and their free energies are (1) on with no ligand bound, 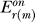 , (2) on with ligand bound, 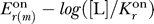 , (3) off with no ligand bound, 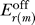 , and (4) off with ligand bound, 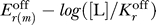 . Here 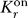 and 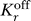 are the binding constants in the on and off states for a specific type of receptor *r*, and *m* is the methylation level (*m* = 0,...,8). Based on experimental data, these binding constants are assumed to be independent of ligand concentration or methylation level [21–23]. For the two on states, the sum of the equilibrium Boltzmann factors is

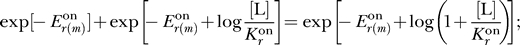
therefore, the combined free energy of the two on states is 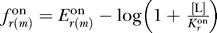 . Similarly, the combined free energy of the two off states is 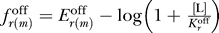 . All energy units are expressed in units of the thermal energy, *k_B_T*. Since binding of attractant favors the off state, *K* ^on^ > *K* ^off^. The opposite applies to repellents: *K* ^on^ < *K* ^off^.

Adaptation to attractant occurs through methylation, which favors the on state. Therefore, the offset energy 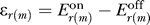 decreases as *m* increases. The free-energy difference between the on and off states of a single receptor is

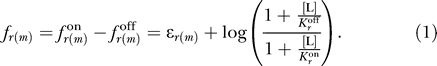



The free-energy difference *F* of a complex of receptors is the summation of the individual *f_r_*_(*m*)_ of all of the receptors in the complex,

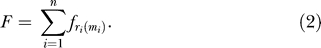



The average activity *A* of the complex of receptors is its probability of being in the on state and is given according to equilibrium statistical mechanics by

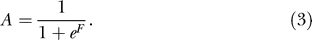



### Receptor Modification by CheR and CheB

Within our model, receptors dynamically bind and unbind the adaptation enzymes CheR and CheB (Figure 1A). We assume that a receptor-bound CheR only methylates receptors when the complex is off, whereas a receptor-bound CheB only demethylates receptors when the complex is on (Figure 1B). For precise adaptation to occur, the rates of methylation and demethylation by CheR and CheB must depend only on the activity of the complex *A*. We assumed each bound CheR adds methyl groups at a rate *k*_R_(1 − *A*), and each bound CheB protein removes methyl groups at a rate *k*_B_ *A*. In most simulations, we assumed saturated kinetics for CheR and CheB. Specifically, we assumed each receptor-bound CheR or CheB acts on available sites in the AN with equal probability, independent of the number of available modification sites. However, we also explored the effects of nonsaturated kinetics by introducing a factor *N*/(*N* + *M*_sat_) into the methylation/demethylation rates, where *N* is the total number of available sites for methylation/demethylation and *M*_sat_ is a constant. Smaller values of *M*_sat_ mean more nearly saturated kinetics, with full saturation corresponding to *M*_sat_ = 0. For all of our simulations, the methylation/demethylation rate is zero if there are no available modification sites (*N* = 0).

Unlike the previous AN model [18,19], we include dynamical CheR/CheB binding and unbinding to receptors. Free receptors bind CheR/CheB molecules at a rate 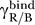 , and receptor-bound CheR/CheB molecules unbind at a rate 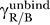 . In addition, each receptor can bind at most one CheR or one CheB. At steady state, this gives for the average proportion of receptors bound by CheR (with a similar expression for CheB):

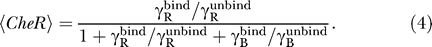



The CheR/CheB binding rates 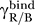 are assumed to increase linearly with the concentration of free CheR/CheB, whereas the CheR/CheB unbinding rates 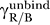 are assumed to be independent of concentration. As the concentration of CheR, and therefore 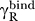 , rises, the proportion of CheR-bound receptors 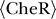 also rises. Since each receptor can bind at most one CheR or one CheB, the proportion of CheR-bound receptors 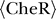 decreases with an increase in the CheB binding rate 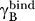 . The CheR/CheB unbinding rates also define an average dwell time each CheR or CheB is bound to a receptor to be 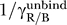 .

A fixed hexagonal arrangement of 19-receptor homodimers (Figure 1A) was used for every simulation. For simplicity, from this point, we refer to each homodimer as a receptor. The ANs consist of a receptor and its nearest neighbors. This creates 19 possible ANs, each centered on one receptor: seven ANs of size 7, six ANs of size 5, and six ANs of size 4. The average AN size is 5.4 receptors. Six receptors were Tar, and 13 receptors were Tsr, consistent with the wild-type ratio [24]. We also explored the effect of AN size through the use of size-one AN complexes and half AN complexes. For size-one AN clusters, each CheR or CheB can only modify the bound receptor. For half AN complexes, we randomly chose half of each receptor's nearest neighbors to be in the AN and used the same configuration for all simulations. For the six receptors with three nearest neighbors, three have half ANs including two adjacent neighbors, and the other three have half ANs including only one adjacent neighbor.

### Mean Field Theory

By considering the average net methylation rate of a complex, we derived a simple mean field theory for complex activity. The average methylation rate of a complex is the rate of methylation by a single CheR times the number of bound CheR with available methylation sites. Similarly, the average demethylation rate of the complex is the rate of demethylation by a single CheB times the number of bound CheB with available demethylation sites. Therefore, the average net methylation rate for a single receptor is

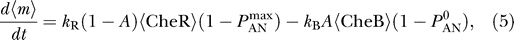
where 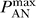 and 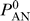 are the average proportions of fully methylated and fully demethylated ANs, respectively. The factors 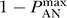 and 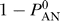 account for the fact that CheR cannot methylate an already fully methylated AN, and CheB cannot demethylate an already fully demethylated AN. The condition that the average net methylation rate is zero (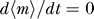 ) determines the average steady-state activity,

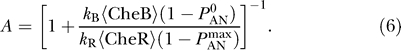



As long as 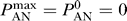 , the activity will always adapt precisely to

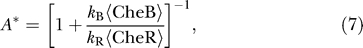
which is independent of ligand concentration or methylation level, since 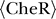 and 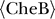 depend only on the concentrations and binding rates of CheR and CheB.

Fluctuations in methylation and demethylation will lead to finite values of 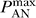 and 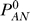 . However, for large enough ANs, the probability that a neighborhood will become fully methylated or fully demethylated by chance will be very small (as long as the average receptor methylation level is not close to 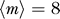 or 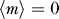 ), so 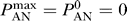 will be a good approximation. Therefore, we expect the activity in large-AN models to adapt to A^*^ over a broad range of ligand concentrations.

As the average receptor-methylation level reaches 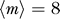 , the methylation level cannot increase to compensate for the increased free-energy difference due to attractant binding. Therefore, activity drops to zero beyond the limiting ligand concentration at which receptors become fully methylated. At full methylation of the complex,

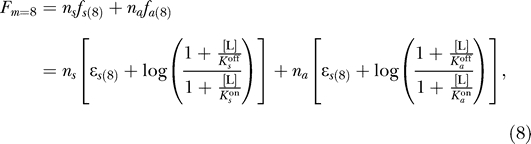
where *n_s_* is the number of Tsr receptors in the complex and *n_a_* is the number of Tar receptors. Therefore, failure of precise adaptation begins near the ligand concentration [L] for which *F_m_*_=8_ = F^*^, i.e., the value of *F* at precise adaptation. Further increase in attractant concentration causes a rapid decay in activity:

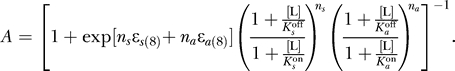



### Analytical Expression for Noise

Here, we derive an analytical expression for the fluctuations of complex activity due to discrete methylation and demethylation events by receptor-bound CheR and CheB. First, we calculate the variance of the total complex methylation level within the Langevin approximation [25]. If the free-energy difference between the active and inactive state of a receptor depends linearly on the methylation level *m*, then for a single receptor

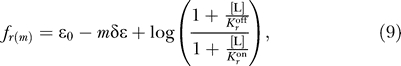
and for a complex of *n_s_* Tsr receptors and *n_a_* Tar receptors (with total methylation level *M*),

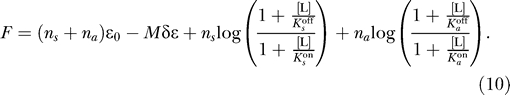



Since CheR/CheB methylation/demethylation rates depend on complex activity, fluctuations in the free-energy difference *F* translate into fluctuations in complex activity *A*. Linearization of Equation 5 with 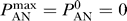 yields for 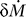 ,

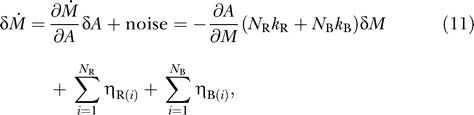
where *N*_R_/*N*_B_ are the number of bound CheR/CheB enzymes, *η*_R(*i*)/B(*i*)_ are independent Langevin noise terms for each bound CheR/CheB, and 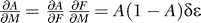 . After a Fourier transform and integration of the power spectrum, we obtain (with 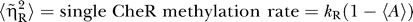 and 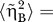 single CheB demethylation rate = 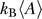 )

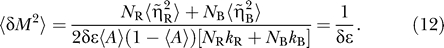



The variance in methylation level depends only (inversely) on *δɛ*, the step in free-energy difference per added methyl group. The variance in activity is therefore

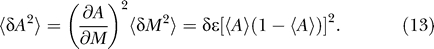



## Results

Figure 2 shows simulated response curves of complexes of 19 chemoreceptor dimers to step increases in concentration of alpha-methyl aspartate (MeAsp), an attractant. The results shown include the dynamics of CheR and CheB (see Model) and are similar to those obtained with static ANs [18]. Precise adaptation occurs over four orders of magnitude of MeAsp concentration, with methylation levels increasing to compensate for drops in activity due to increases in attractant concentration.

**Figure 2:**
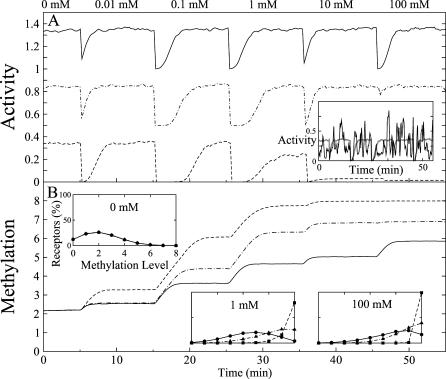
Averaged Response Curves for Step Increases in Attractant Concentration (0–100 mM) A mixed receptor complex of six Tar and 13 Tsr receptors (solid curve) is compared to complexes of 19 Tar receptors with 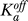 = 0.06 mM (dot-dashed curve) and 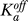 = 0.02 mM (dashed curve). Other parameters are given in the Model section. Simulations were averaged over 500 runs. (A) Averaged response of complex activity *A* (Equation 3). Upper curves are each displaced vertically by 0.4. Inset: response curve for one simulation of a single mixed complex. The average activity is superimposed in gray. (B) Averaged methylation *m* of receptor homodimers. Insets: distribution of methylation levels at 0 mM, 1 mM, and 100 mM.

In Figure 2, the Tar-only complexes exemplify two different limits of precise adaptation at high attractant concentrations, as in the static AN model [18]. For the Tar-only complex with higher 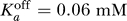 (dot-dashed curve), the activity continues to adapt precisely, but the activity stops responding to increases of MeAsp. In this case, the receptors become saturated, and further increases in MeAsp do not produce changes in the free-energy difference between the on and off states of the complex. The average methylation of the complex reaches a constant value, below full methylation (Figure 2B). In contrast, for the complex with lower 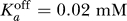 (dashed curve), the activity approaches zero at high concentrations. In this case, full methylation occurs before saturation of the receptors with MeAsp. When MeAsp concentrations increase further, the resulting increase in the free-energy difference between the on and off state of the complex cannot be compensated by additional methylation, so the activity drops without recovering.

Compared to the Tar-only complexes, the heterogeneous receptor complex with six Tar and 13 Tsr receptors (solid curve) continues to respond to MeAsp increases and to adapt precisely over an extended range. The Tar receptors in this complex have 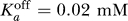 , as in the second case considered above (dashed curve); these six Tar receptors allow for a sensitive response at low concentrations of MeAsp. In contrast, the 13 Tsr receptors in the complex have low affinity for MeAsp. Therefore, at low MeAsp concentrations the Tsr receptors act as extra methylation sites, increasing the range of precise adaptation. As MeAsp concentrations increase, the Tar receptors become fully saturated, but the Tsr receptors begin to bind MeAsp. This increases the upper limit of response to well over 100 mM MeAsp.

For homogeneous complexes, the limit of adaptation at high attractant concentration depends on which occurs first, saturation of receptors by attractant or full methylation of receptors. Which of these occurs first depends on the ratio 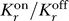 . The crossover ratio between the two limits of adaptation can be estimated in mean field theory (Equation 2). At high ligand concentrations:

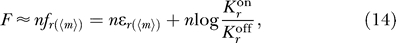
where *n* is the number of receptors in the complex. Loss of activity occurs if the offset energy at full methylation *ɛ_r_*_(8)_ cannot compensate for the free-energy difference per receptor due to saturating attractant, 
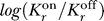
 . Therefore, for F^*^ denoting the precisely adapted free-energy difference, if

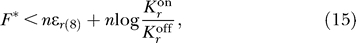
or equivalently, if 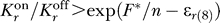 , loss of activity will occur at high concentrations of attractant (dashed curve in Figure 2A). In contrast, if 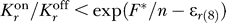 , loss of response will occur at high concentrations of attractant (dot-dashed curve in Figure 2A). For fixed *ɛ_r_*_(*m*)_, F^*^, and complex size *n*, the limit of adaptation depends only on the ratio 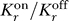 , not on the individual magnitudes of 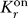 and 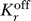 . For our simulation, *ɛ_r_*_(8)_ = −30, *n* = 19, and A^*^ = 1/3, so F^*^ = log2 = 0.693. Therefore, the expected crossover ratio is exp(F^*^/*n* − *ɛ_r_*_(8)_) = 20.8. For Tar-only complexes with 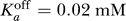 , 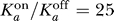 , so adaptation fails through loss of activity as observed in Figure 2A (dashed curve). For Tar-only complexes with 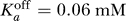 , 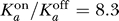 , and adaptation fails through loss of response, as also observed in Figure 2A (dot-dashed curve).

Experiments indicate that the adapted tumbling rate, and therefore, also the adapted receptor activity, increases with the concentration of CheR [26]. At low levels of CheR, the binding rate 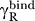 is proportional to the concentration of CheR. In Figure 3, we show the adapted activity as a function of the CheR binding rate 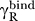 . Adapted activity is the average activity calculated according to Equation 3, after allowing the complex to reach equilibrium (see Methods). As the binding rate of CheR increases, the proportion of CheR-bound receptors 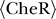 also increases (Figure 3, inset). The increase in 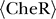 causes the rate of methylation for the whole complex to rise, therefore increasing activity. The complex with full ANs (including all nearest neighbors) closely follows the expected mean-field-theory result (Equation 7), whereas the complex with ANs of size one deviates to higher activity over a wide range of CheR binding rates. In these simulations, no attractant is present, and therefore, the average methylation level of the receptors is low. Consequently, complete demethylation of individual receptors is likely to occur in the AN = 1 model (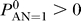 ), leading to missed demethylation attempts, and therefore, to an increase in adapted activity according to Equation 6. In effect, for the AN = 1 model, the demethylation rate is lower than it “should be” because by chance, some individual receptors are already fully demethylated, and therefore, CheB fails to act sometimes when it “should.”

**Figure 3:**
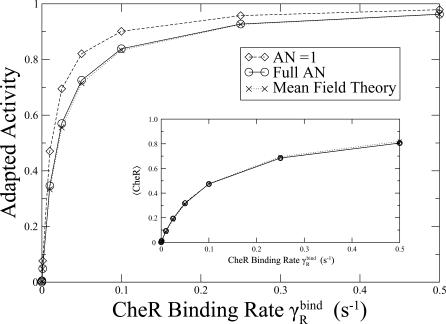
Adapted Activity as a Function of CheR Binding Rate 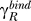 Results are shown for ANs of size one, ANs of all adjacent receptors, and the mean field theory. The other enzyme binding rates are 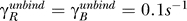 and 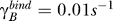 . Inset: 〈CheR〉 (proportion of receptors bound to CheR) as a function of CheR binding rate.

In Figure 4, we explore precision of adaptation over a broad range of MeAsp concentrations for several variants of our model. In general, deviations from precise adaptation occur if and only if the rates of methylation or demethylation cease to depend exclusively on complex activity (Equation 6). We find that large AN sizes, saturated kinetics of CheR/CheB, and short CheR/CheB dwell times favor precise adaptation. In all cases, we consider the same complex of 19 receptors (Figure 1A) composed of six Tar receptors and 13 Tsr receptors. For comparison, we also show the mean field theory result (see Model).

**Figure 4:**
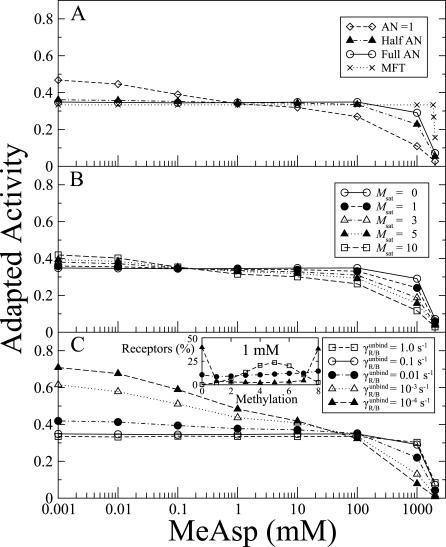
Precision of Adaptation The adapted activity is shown as a function of MeAsp concentration for variants of the model. (A) Various AN sizes (and mean field theory). For “Half AN,” the assistance neighbors of each receptor were chosen at random (see Model). (B) Different saturation factors *M*_sat_, for both CheR and CheB. The rate of methylation/demethylation varies as *N*/(*N* + *M*_sat_), where *N* is the number of available modification sites. (C) Different binding/unbinding rates for2 CheR and CheB. The ratio 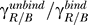 was kept constant at 10. Inset: distribution of receptor methylation levels at 1 mM MeAsp for 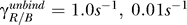 , and 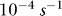 10^−4^ s^−1^.

In Figure 4A, we show the effect of AN size on precise adaptation. Within each AN, there is a “ladder” of possible methylation levels. Fluctuations cause the methylation level to move up and down the ladder, deviating from the average. For small ANs, the ladder is shorter, and fluctuations are more likely to produce fully methylated or fully demethylated neighborhoods. At low levels of MeAsp and low average methylation, fluctuations are likely to produce fully demethylated neighborhoods, lowering the rate of demethylation and increasing activity according to Equation 6. Similarly, at high levels of MeAsp and high average methylation, neighborhoods may become fully methylated, lowering the rate of methylation by CheR and decreasing activity.

As shown in Figure 4A, complexes with ANs of size one have a drastically reduced precision of adaptation, but half neighborhood complexes have a precision of adaptation close to that of full AN complexes. Beyond a certain AN size, the methylation ladder is already long enough to effectively prevent fluctuations from causing full methylation or full demethylation of neighborhoods. Therefore, increasing AN size improves precision of adaptation only up to a point, beyond which AN size only affects activity near the concentration at which all receptors become fully methylated. For our parameters, receptors do not become fully demethylated even at zero attractant concentration, but full demethylation could be induced by addition of repellent.

We also performed simulations with varying degrees of saturation of CheR and CheB (Figure 4B). Specifically, we introduced a factor of *N*/(*N* + *M*_sat_) into the rates of CheR and CheB action, where *N* is the total number of available sites for methylation/demethylation and *M*_sat_ is a constant (see Model). For all other simulations, CheR and CheB were assumed to work at saturation, independent of methylation level (*M*_sat_ = 0). Increasing *M*_sat_ makes the rate of action of CheR and CheB more dependent on the number of available modification sites. For finite *M*_sat_, in low concentrations of MeAsp, and therefore, low average methylation levels, the rate of demethylation by CheB is significantly lower than the saturated (maximal) rate. Conversely, there are many available sites for methylation, so the rate of methylation is near maximal. Therefore *N*_B_/(*N*_B_ + *M*_sat_) < *N*_R_/(*N*_R_ + *M*_sat_) ≈ 1. A relative decrease in the rate of demethylation by CheB compared to the rate of methylation by CheR causes an increase in the activity of the complex as seen below 0.1 mM MeAsp in Figure 4B. As the average methylation level increases with increasing MeAsp concentration, the inequality is reversed so that *N*_R_/(*N*_R_ + *M*_sat_) < *N*_B_/(*N*_B_ + *M*_sat_) ≈ 1 results in a relative decrease in the rate of methylation by CheR compared to the rate of demethylation by CheB. Therefore, at high MeAsp concentration, above 0.1 mM, the adapted activity decreases below the expected value for precise adaptation.

Within mean field theory for *M*_sat_ > 0, we can approximate the crossover concentration, i.e., the concentration of attractant at which the activity of complexes is equal to the expected precisely adapted activity. The crossover occurs when the saturation factors of CheR and CheB are equal, *N*_B_/(*N*_B_ + *M*_sat_) = *N*_R_/(*N*_R_ + *M*_sat_). This occurs when the average methylation level 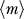 is 4, which occurs at 0.36 mM MeAsp. This is close to the crossover concentration observed in our simulations (Figure 4B).

Simulations were also performed in which the average dwell time of CheR and CheB was varied (Figure 4C). The average dwell time is equal to 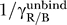 , whereas the average number of enzymes bound to the complex depends on the ratios 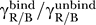 (Equation 4). Therefore, in order to change the average dwell time while conserving the average number of CheR and CheB enzymes bound to the complex, we altered both 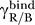 and 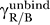 by the same factor. In the model, when a CheR or CheB is bound for a long time, the enzyme catalyzes the same reaction numerous times before unbinding. The methylation level in the neighborhood will therefore move along the ladder in one direction, possibly reaching the end, i.e., full methylation or full demethylation. As for the AN = 1 model in Figure 4A, the result in Figure 4C for long CheR and CheB dwell times is higher activity at low MeAsp concentrations (where full demethylation is more likely) and lower activity at high MeAsp (where full methylation is more likely).

Deviations from mean field theory occur if the average dwell time of CheR or CheB is long enough to allow full methylation or demethylation of neighborhoods. Below 0.001 mM MeAsp, the average adapted methylation level per receptor homodimer is ≈ 2.2. Since there are on average 5.4 receptors per AN, the average distance to the bottom of the methylation ladder is 2.2 × 5.4 ≈ 12. Therefore, precise adaptation is expected to fail when the demethylation rate is ≈12 times the CheB unbinding rate (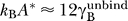 ). For our parameters, A^*^ = 1/3 and *k*_B_ = 0.2 s^−1^, we expect precise adaptation to fail for 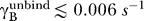 . Consistent with this calculation, our simulations show that deviations from precise adaptation begin to occur at low MeAsp concentrations for 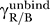 around 0.01 s^−1^.

The fact that most receptors are either fully methylated or fully demethylated for long dwell times of CheR and CheB is clearly shown by the distribution of methylation levels for different average dwell times (Figure 4C, inset). As dwell time increases, the single-peaked methylation distribution flattens out and becomes bimodal, i.e., most receptors become fully methylated or fully demethylated. Addition of ligand causes a shift in the amplitudes of the two peaks, but the peak positions, at *m* = 0 and *m* = 8, do not change. We can exploit this fact along with mean field theory to estimate the crossover attractant concentration where the activity of the complex crosses A^*^. The average methylation (demethylation) rate has a correction factor equal to the proportion of not fully methylated (not fully demethylated) ANs (Equation 6). The crossover attractant concentration will occur where these two correction factors are equal, namely where 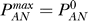 , which implies 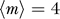 . For our mean field–adapted activity of A^*^ = 1/3, and requiring 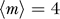 , we obtain a crossover concentration of 30 mM MeAsp, consistent with the simulation results shown in Figure 4C.

In all our simulations, the methylation levels of receptors fluctuate, translating into fluctuations in complex activity. Figure 5 shows the distribution of activities due to fluctuating methylation levels at 0 mM, 1 mM, and 100 mM of MeAsp. Within the MWC model, complex activity is strictly either zero or one. However, we assume that switching between these two states is rapid, so we consider the distribution of thermally averaged complex activities given by Equation 3. Even for the full AN model, for which adaptation is precise, there is a broad range of complex activities. Note though that for the observed variation in activity of ≈50% for a single complex and assuming ≈500 independent receptor complexes per cell, the resulting variation in total activity would be only ≈2.5%. As shown in Figure 5, for size-one ANs at 0 mM and 100 mM MeAsp, the activity distributions are shifted relative to the activity distributions for full ANs because adaptation is not precise when CheR and CheB act only on single bound receptors (cf. Figure 4A). Also shown in Figure 5, long dwell times of CheR and CheB cause a bimodal distribution of complex activities, corresponding to the bimodal distribution of receptor methylation levels (cf. Figure 4C, inset).

**Figure 5:**
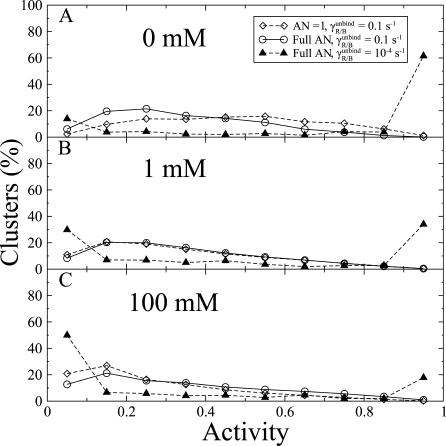
Distribution of Adapted Complex Activities (Reflecting Distribution of Complex Methylation Levels) at Different MeAsp Concentrations The MeAsp concentrations are as follows: (A) 0 mM, (B) 1 mM, and (C) 100 mM. Distributions are shown for AN = 1 and full AN at 
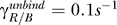
 , and for full AN at 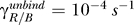 . The ratio 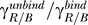 was kept constant at 10.

Within our model, noise is caused by fluctuations in both binding/unbinding of CheR and CheB and methylation/demethylation by CheR/CheB. For short average dwell times, fluctuations in the number of bound CheR and CheB enzymes are rapidly averaged out, and the dominant source of noise is the discrete methylation/demethylation events by receptor-bound CheR/CheB. We have estimated the resulting variance in complex methylation 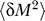 and activity 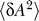 with the linear noise approximation (see Model and Figure 6). In this limit, the only factor that affects the variance 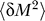 is the free-energy difference *δɛ* per methyl group, with 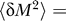 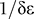 . In the opposite limit of long average dwell times, fluctuations in the number of CheR and CheB enzymes bound to the complex add to the variance in methylation levels and thus activity. As seen in Figure 6A and 6C, low binding and unbinding rates 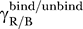 cause an increase in noise over the calculated theoretical noise limit due to the discreteness of methylation and demethylation events. Increasing complex size can decrease the noise due to CheR and CheB binding/unbinding, but not the noise due to CheR/CheB methylation/demethylation. Therefore, increasing complex size only decreases noise for long average dwell times of CheR and CheB, but has no effect in the case of short dwell times, where noise is near the theoretical limit (Figure 6B and 6D).

**Figure 6:**
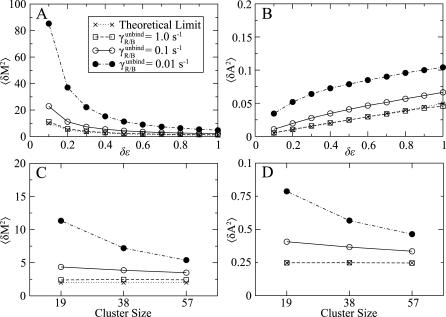
Variance in Complex Methylation and Activity Levels Curves are shown for CheR/CheB binding/unbinding rates 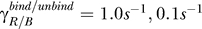 , and 0.01 s^−1^, as well as for the theoretical limit from the linear noise approximation (see Model). (A,C) Variance in complex methylation level as a function of the free-energy step *δɛ* per methyl group (A) and complex size (C) (see Methods). (B, D) Variance in complex activity level as a function of *δɛ* (C) and complex size (D).

It was observed experimentally by Chalah and Weis [27] that CheR and CheB methylate/demethylate the four different methyl-attachment sites on each receptor monomer at different rates. These observations suggest two possible scenarios: either CheR and CheB have different rates of action on different modification sites, or CheR and CheB divide their time unequally among the sites (or some combination of these two). To test the first scenario, we extended our model to include variation in the rates of action of CheR and CheB, with the results shown in Figure 7. Specifically, we assumed that when a CheR or CheB is tethered to a receptor, it divides its time equally among all available modification sites in the AN. The total rate of action by a bound CheR or CheB is therefore the average over the rates for all available modification sites in the AN. The catalytic rates for a methylation/demethylation reaction were assumed to vary in the ratio 1:2:4:8 for the four different sites [27]. We studied two cases. In the first case, the ratios of methylation and demethylation matched for each site (i.e., for sites 1–4, the ratios for both *k*_B_ and *k*_R_ were 1:2:4:8). In the second case, the ratios for methylation and demethylation were inverted relative to each other (i.e., for sites 1–4, the ratios for *k*_B_ are 1:2:4:8 and for *k*_R_ 8:4:2:1).

**Figure 7:**
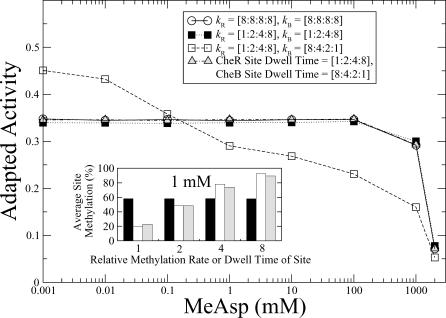
Precision of Adaptation for Receptors with Site-Dependent and Site-Independent Methylation/Demethylation Rates Shown are site-dependent methylation/demethylation rates with matching ratios (*k*_R_ = 0.0125,0.025,0.05,0.1 s^−1^; *k*_B_ = 0.025,0.05,0.1,0.2 s^−1^), with inverted ratios (*k*_R_ = 0.0125,0.25,0.05,0.1 s^−1^; *k*_B_ = 0.2,0.1,0.05, 0.025 s^−1^), for receptors with site-independent methylation/demethylation rates (*k*_R_ = 0.1 s^−1^; *k*_B_ = 0.2 s^−1^), and for receptors with site-dependent CheR dwell times in the ratio 1:2:4:8 and inverted site-dependent CheB dwell times in the ratio 8:4:2:1. Inset: average site methylation at 1 mM MeAsp for receptors with site-dependent methylation/demethylation rates for both the matching-ratio case (filled bars) and the inverted-ratio case (open bars), and for receptors with inverted site-dependent CheR/CheB dwell times (gray bars).

As shown in Figure 7, when the ratios of methylation and demethylation match for each site, precise adaptation is preserved. In this case, since every site has the same ratio of *k*_B_/*k*_R_ as every other site, the average methylation levels of all sites remain the same, as shown in the inset. The average methylation and demethylation rates over sites is therefore constant, independent of ligand concentration, preserving precise adaptation. In contrast, inverted ratios of methylation and demethylation rates among the sites fail to produce precise adaptation. In this case, the ratio *k*_B_/*k*_R_ varies among the four methylation sites, causing varying equilibrium methylation levels (Figure 7, inset). The sites with a low *k*_B_/*k*_R_ ratio are the first to become methylated at low concentrations of MeAsp, leading to low average rates of demethylation compared to methylation, and therefore to high adapted activity. As average methylation levels rise with increasing MeAsp, these low *k*_B_/*k*_R_ sites “fill up,” leading to high average rates of demethylation compared to methylation, and therefore to low adapted activity.

The second scenario suggested by the Chalah and Weis data [27], namely different dwell times for CheR and CheB among the modification sites, leads more robustly to precise adaptation. As long as CheR and CheB work near saturation, differences in dwell times between sites will not affect total rates of methylation and demethylation, and precise adaptation will be preserved, according to Equation 6. Indeed, as shown in Figure 7, even if the relative dwell times for each site are inverted for CheR and CheB, precise adaptation is preserved.

Experiments by Berg and Brown [9] on wild-type E. coli indicate that whereas adaptation to aspartate is precise over a large concentration range, precise adaptation to serine fails at relatively low concentrations. In Figure 8, we compare our model to these experiments. In both cases, adaptation to aspartate (or MeAsp) is precise over four orders of magnitude. However, adaptation to serine fails at approximately 0.1 mM. Within our model, this difference with respect to attractants reflects the presence of more Tsr receptors (13) in the complex than Tar receptors (six). More Tsr receptors amplify the change in complex free energy due to serine, which results in an increased sensitivity at low concentrations, but also results in full methylation of the complex and loss of activity beginning at 0.1 mM serine.

**Figure 8:**
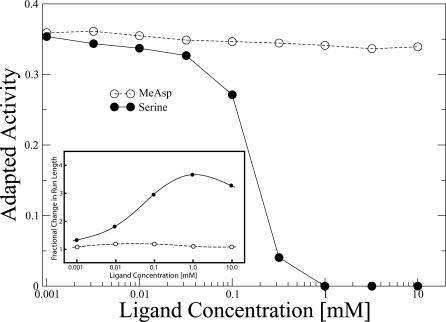
Adapted Complex Activity versus Concentration of MeAsp (Filled Circles) or Serine (Open Circles) The saturation factor is *M*_sat_ = 1, and other parameters are given in the Model section. Setting *M*_sat_ = 0 would sharpen and shift the drop in activity to higher serine concentration [18]. Inset: experimental measurement of the fractional change in run length versus concentration of aspartate (open circles) or serine (closed circles) [9].

We tested robustness of our theoretical model by randomly varying parameters as described in the Model section. The results shown in Figure 9 demonstrate that precise adaptation is a robust property of our model. Almost ideal adaptation occurs for all parameter sets up to a total parameter variation of *K* ≈ 10^18^. For larger parameter variations, in the range of *K* = 10^4^–10^5^, 77% of the altered models still have a precision of adaptation within 10%. These results are similar to those obtained from the simple single-receptor model of Barkai and Leibler [16]. However, in one regard, our MWC model with ANs is more robust than the single-receptor model. In the single-receptor model, precise adaptation requires that the activity of the receptor is zero at full demethylation and one at full methylation. Our model has the property of precise adaptation without this assumption.

**Figure 9:**
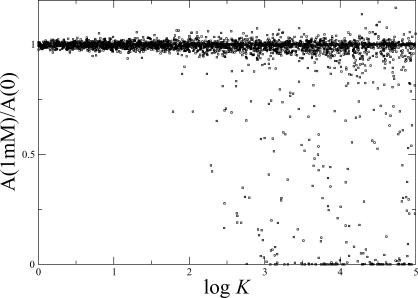
Robustness of Assistance-Neighborhood Model for Adaptation Ratio of adapted activity at 1 mM MeAsp to adapted activity at 0 mM MeAsp plotted as a function of total parameter variation 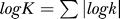 (see Methods). The scatter plot shows results for 3,000 different parameter sets.

## Discussion

The chemotaxis system in the bacterium E. coli is remarkably sensitive to small relative changes in the concentrations of multiple chemical signals over a broad range of ambient concentrations. We have presented a model of complexes of strongly coupled chemoreceptors to account for precise adaptation, as well as other properties of the chemotaxis network. Similarly to the BBL model of precise adaptation for a single two-state receptor, CheR only methylates inactive receptors, and CheB only demethylates active receptors [16]. A previous MWC model [18,19] extended the BL model to fit observations of in vivo receptor clustering [10,11] and of the action by CheR and CheB on ANs of five to seven adjacent receptor homodimers [15]. Our model builds upon this earlier MWC model, but includes dynamic ANs, created by the transient binding of CheR and CheB to tethering sequences at the C termini of receptors [14]. The importance of tethering of enzymes has recently attracted considerable theoretical interest [28–31]. Transient CheR and CheB binding is of particular relevance because the experimentally observed ratio of receptor homodimers to the enzymes CheR and CheB is approximately 50:1 and 30:1, respectively [4]. Therefore, receptors are not likely to be continuously in the AN of an CheR or CheB enzyme. Here, we have shown that a model with dynamical CheR/CheB binding and unbinding to receptors can reproduce precise adaptation as in the previous AN model [18]. However, CheR/CheB dynamics can both limit precise adaptation and increase noise in complex activity. In addition, we have expanded our results to show robust adaptation, to explain the experimentally observed difference between the responses to aspartate and serine, and to account for the persistence of precise adaptation despite the experimentally observed kinetic variation among methylation sites.

Although both MWC models [11,18–20,32] and Ising-type lattice models [33–35] have been used to represent interactions among receptors, analysis of FRET data provides evidence for strongly coupled MWC complexes [36]. Mello and Tu [20,32] successfully fit the Sourjik and Berg FRET data [10] using an identical MWC model to ours [18,19], but did not include CheR/CheB kinetics. Although Mello and Tu considered methylation-dependent ligand-binding constants *K* ^on/off^, fitting results do not require variable *K* ^on/off^. CheR and CheB dynamics have been explored in a mixed-receptor Ising-type lattice model by assuming Michaelis-Menten kinetics, with each CheR or CheB only able to methylate or demethylate the bound receptor once before detaching [34,35]. In principle, combining catalysis with unbinding increases precision of adaptation by decreasing the likelihood of fully methylating or fully demethylating a receptor, but enzyme tethering suggests each bound enzyme may catalyze multiple methyl transfers before unbinding.

Within our model, deviations from precise adaptation occur only if CheR/CheB methylation/demethylation rates become dependent on the receptor-methylation level. Small AN size and long dwell times of CheR and CheB cause full methylation or demethylation of neighborhoods, resulting in methylation-dependent rates and failure of precise adaptation. Although the average dwell time of CheR or CheB has not been experimentally determined, the diffusion-limited association rate of protein–protein interactions is on the order of 10^5^–10^7^ M^−1^s^−1^ [28,37]. Multiplying by the experimentally determined dissociation constant of 11 μM for CheR [38] yields unbinding rates in the range of 1–100 s^−1^, well above the CheR unbinding rate 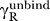 required for precise adaptation (Figure 4). Precise adaptation also requires saturated enzyme kinetics, meaning that each bound CheR or CheB acts at a rate independent of the number of available modification sites. (Unsaturated kinetics would imply decreased demethylation rates at low average methylation levels, and decreased methylation rates at high average methylation levels.) In our model, precise adaptation is robust to differences in dwell times of CheR or CheB on different modification sites, but not, in general, to different rates of CheR or CheB action on these sites, pointing to different dwell times as the explanation for the site-dependent methylation/demethylation rates observed by Chalah and Weis [27].

As with the BL model for a single receptor, our model for a receptor complex robustly yields precise adaptation over a wide range of parameters (Figure 9). One improvement is that our model based on ANs does not require fully methylated receptors to be fully active, or fully demethylated receptors to be fully inactive. Experiments indicate that adaptation time and adapted activity level vary even among genetically identical cells [39]. Consistent with observations by Alon et. al. [26], our model predicts that varying CheR and/or CheB concentrations will lead to different adapted activities (Figure 3) while preserving precise adaptation. The robustness of the essential properties of the network (e.g., sensitivity and precise adaptation) presumably also allows for genetic polymorphisms in the binding and reaction rates of network proteins, making the network robust to evolutionary change.

Within our model, we assume that the rates of modification by CheR and CheB depend directly on complex activity. In fact for precise adaptation, only one enzyme needs to respond directly to complex activity. This is consistent with experiment as CheR rates are affected directly by activity [40], whereas CheB is phosphorylated to an active form by the receptor-regulated kinase CheA [41,42], implying a global feedback mechanism. If, hypothetically, all feedback were global, there would be no direct “return force” on the activity of individual complexes, only an indirect return force on the average complex activity. As a result, sensitivity would be lost as most complexes would drift to nonresponsive methylation levels, becoming either fully active or fully inactive. However, within our model, precise adaptation still occurs if the CheB feedback mechanism is disabled without destroying CheB's demethylating ability as long as direct feedback from complex activity to CheR is maintained. Indeed, experiments mutating the phosphorylation site of CheB demonstrate that CheB phosphorylation is not required for precise adaptation [26], but is important to keep adapted CheY-P levels in the range of motor sensitivity [43].

Our model helps explain the advantage of multiple methylation sites per receptor. First, the number of receptors that a tethered CheR or CheB can modify is constrained by the physical length of the tether. Therefore, to provide enough steps in the ladder of methylation levels to prevent full demethylation or full methylation of neighborhoods (and therefore loss of precise adaptation), the number of modification sites per receptor must be sufficiently large. Second, if the number of methylation sites per receptor were small, then to allow precise adaptation over a large concentration range would require a large change in free energy per methyl group. However, large free-energy steps per methyl group increase the noise in activity (Figure 6), and prevent complexes from operating in the regime of maximal sensitivity.

One longstanding puzzle has been the observed difference in E. coli's chemotactic response to serine and aspartate [9]. Our model explains both the observed broad range of precise adaptation to aspartate/MeAsp and the failure of adaptation at relatively low serine concentrations (Figure 8). Based on receptor in vivo expression levels, complexes contain more Tsr receptors then Tar receptors, so the Tsr receptors act as extra methylation sites and increase the range of precise adaptation to aspartate/MeAsp. As the Tar receptors become fully saturated, the Tsr receptors bind aspartate/MeAsp, thereby also broadening the range of response. In contrast, the high proportion of Tsr receptors amplifies the complex free-energy change due to serine and leads to full methylation of receptors and, therefore, loss of activity, beginning at 0.1 mM serine. The prevalence of Tsr receptors suggests that chemotaxis to low concentrations of serine is biologically important.

For stimulation of low-abundance (minor) receptors, our model predicts a limited range of response. With approximately one minor receptor of each type per complex, there is no amplification of free-energy change, so sensitivity is limited to the off-state ligand affinity 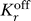 . The range of response is then constrained by the on-state ligand affinity 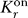 , unless the range of response is extended though weak binding of ligand to other receptors.

The effect of AN size may be testable experimentally through shortening the flexible tether at the C terminus of receptors while preserving the pentapeptide binding site for CheR and CheB. Decreasing neighborhood size should produce deviations from precise adaptation as the ends of the methylation ladders for ANs are reached (Figure 4A). In addition, the consequence of nonsaturated kinetics may be testable through mutations in CheR/CheB that reduce their affinities for the methyl-modification sites on receptors. Our model predicts global failure of precise adaptation for large deviations from fully saturated kinetics, but even small deviations from full saturation have noticeable consequences near full methylation (Figure 4B).

Experiments demonstrate that precise adaptation is a robust property of the E. coli chemotaxis network [26]. The elegant BL model exhibits robust adaptation through integral feedback control [44], but does not include interactions among receptors. Our model provides a molecular mechanism illustrating how integral feedback control is implemented in the presence of receptor clustering, and highlights the importance of ANs to effectively increase the ladder of methylation levels.

## Methods

### Model parameters.

In calculating complex activity, we used the same offset energies for both Tar and Tsr receptors: *ɛ_r_*_(0)_ = 1.0; *ɛ_r_*_(1)_ = 0.5; *ɛ_r_*_(2)_ = 0.0; *ɛ_r_*_(3)_ = −0.3; *ɛ_r_*_(4)_ = −0.6; *ɛ_r_*_(5)_ = −0.85; *ɛ_r_*_(6)_ = −1.1; *ɛ_r_*_(7)_ = −2.0; and *ɛ_r_*_(8)_ = −3.0. Both MeAsp and serine were considered as attractants. For MeAsp (Tar = *a* and Tsr = *s*): 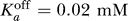 , 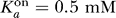 , 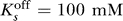 , 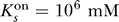 . For serine: 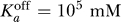 , 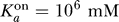 , 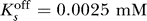 , 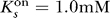 . The constants chosen for Tar-MeAsp binding and Tsr-serine binding are approximately consistent with experimental data [10,45]. The high values of 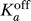 and 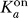 for serine indicate that Tar does not bind serine at the concentrations considered (≤2 M). On the other hand, Tsr binds MeAsp at lower concentrations since 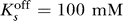 . Both MeAsp and serine are attractants, so *K* ^on^ > *K* ^off^. For the demethylation rate, we used the (rounded-off) observed value *k*_B_ = 0.2 s^−1^ [46,47], and for the methylation rate we set *k*_R_ = 0.1 s^−1^. Since *k*_B_ = 2*k*_R_, this sets an adapted activity of 1/3, assuming that the bound levels of CheR and CheB are the same. Unless otherwise specified, we used the same rates of binding/unbinding for CheR and CheB: 
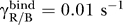
 and 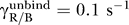 , yielding 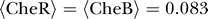 .

### Simulation algorithm.

To simulate the dynamics of an MWC complex of receptors, we used an exact stochastic Gillespie algorithm [48]. We assumed that the rates of ligand binding/unbinding and on/off switching of complexes are much faster than the rates of receptor modification and the rates of CheR/CheB binding and unbinding. Therefore, methylation/demethylation and CheR/CheB dynamics were modeled explicitly, whereas the average activity of the complex was calculated using Equation 3. The Gillespie algorithm involves three different steps for the generation of each data point. First, the reaction that occurs is picked randomly, with weighting directly proportional to the individual rates of each event. The possible events are methylation, demethylation, binding of CheR or CheB, and unbinding of CheR or CheB. A receptor cannot have both CheR and CheB bound at the same time. Next, the site of the event is randomly chosen. The time is then incremented by *τ* = −(log *r*)/Γ, where *r* is a random variable picked from a uniform distribution over [0,1], and Γ is the sum of the rates of all possible events.

For each attractant concentration, simulations to determine adapted activity and distributions (Figures 3–8) were averaged over 200 runs of 10,000 Gillespie steps, each following 10,000 steps to allow time for equilibration.

### Robustness.

In order to test the robustness of our dynamical model, we randomly varied the parameters and tested the precision of adaptation. Altered systems were obtained by modifying eight parameters (*k*_R_, *k*_B_, 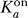 , 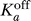 , 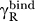 , 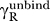 , 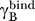 , and 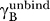 ) by factors of *k_n_*_=1,...,8_. Total parameter variation is expressed by 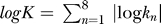 . Each *K* was randomly chosen as *K* = 10^5*r*^, where *r* is a random variable picked from a uniform distribution over [0,1]. Values of the |log *k_n_*| were randomly chosen over [0,1], and were then normalized to yield the correct sum for log *K*. The sign of each log *k_n_* was then chosen with equal probability to be negative or positive. These systems were then subject to a concentration change from 0 mM of ligand to 1 mM. Precision of adaptation was calculated by dividing the adapted activity at 1 mM by the adapted activity at 0 mM.

### Noise.

Simulations to test the effect of the free-energy difference *δɛ* per methyl group on the variances in methylation and activity levels (Figure 6) were performed at 10 mM of MeAsp, with the constant free-energy offset *ɛ*_0_ set to yield an average adapted receptor methylation level of 〈*m*〉 = 4 (i.e., *ɛ*_0_ = −1.0 + 4*δɛ*). *δɛ* = 0.5 was chosen to approximate the free-energy difference per methyl group used in all other simulations. For increased complex sizes, we used two or three strongly coupled 19-receptor complexes (i.e., all receptors on or off together) to produce complexes of size 38 or 57, respectively, preserving the original AN pattern.

## Supporting Information

### Accession Numbers

The primary protein accession numbers (in parentheses) from the Swiss-Prot databank (http://www.ebi.ac.uk/swissprot) for the proteins mentioned in the text are as follows: CheA E. coli CHEA_ECOLI (P07363), CheB E. coli CHEB_ECOLI (P07330), CheR E. coli CHER_ECOLI (P07364), CheW E. coli O157 CHEW_ECO57 (P0A966), CheY E. coli O157 CHEY_ECOLI (P0AE67), Tar E. coli MCP2_ECOLI (P07017), and Tsr E. coli MCP1_ECOLI (P02942).

## Abbreviations

AN: assistance neighborhood
BL: Barkai–Leibler
FRET: fluorescence resonance energy transfer
MeAsp: alpha-methyl aspartate
MWC: Monod–Wyman–Changeux
